# Purifying the Skin

**Published:** 1875-02

**Authors:** 


PURIFYING THE SKIN.—Man is, take him all around, a very dirty animal. The amount of fluid formed by the sweat glands underneath the skin is ordinarily about two pounds, or two pints in the day upon the whole body when the person is comparatively at rest. If exercise is taken to any great extent much more of this secretion or perspiration is thrown off. When the skin is viewed with a magnifying glass of moderate power, it is seen that minute particles of moisture ooze from the pores, which speedily evaporate. The object of perspiration is to regulate the temperature of the body. It is found, in connection with the circulation of the blood, that there is a process going on by which oxygen is being taken out of the air by the arterial blood, and that carbonic acid is being taken away by the veins. The heat of the body is constantly being formed. The normal temperature of the blood is about ninety-eight degrees, and were it not for the process of perspiration, which cools the system, we would get a temperature that would be inconsistent with life. The temperature of the body cannot be sustained ten degrees above or below its normal temperature without life ceasing. It will then be seen that the limits of temperature within which life can be sustained lies within a very narrow range, and hence the importance of perspiration by which the temperature of the body is regulated. In ancient times the first act of hospitality shown to a visitor was to prepare a bath, as mentioned in the case of Ulysses and Telemachus whenever they were hospitably received in their travels. In Greece the bath was both a public and private institution. In Athens the practice of bathing was joined with that of the gymnasium, and Plato recognized the necessity of bathing so much that he made the bath one of the institutions of the State. When the explorations were made in Pompeii in 1824 the ruins of baths were found similar to those that existed in Rome, covering 10,000 square feet. The Romans were in the habit of bathing seven or eight times in the day. In modern times the Turks had cultivated the use of the bath more than any other nation. Mohammed ordained that there should be prayer five times a day with ablutions, and it is clear that however much we pray, we bathe much less than this. In India the bath had always been associated with shampooing It might be supposed that a person getting into such a perspiration as the Turkish Bath induces, and exposed to such extremes of temperature, would be in danger of taking cold. But there is nothing whatever to be feared upon this account. Russian noblemen heat certain rooms in their houses with steam, to a temperature of from 112 deg. to 120 deg. and then pass into a cold bath ; and their serfs, for want of similar accommodation, after taking the steam bath, jump through the window and plunge themselves in the snow. These baths are there considered beneficial to all persons in moderate health and are dangerous only to persons suffering from heart disease. A cold bath is one in which the temperature ranges from 32 deg. to 60 deg, and the warm bath from 98 deg. to 112 deg. There are two classes of bathers, the ducks and the chickens, or those who are continually bathing themselves and those who seldom bathe at all. Too much bathing is injurious to health, but the judicious use of the bath is highly beneficial. It is to be hoped the day is not far distant when the authorities of this and other large cities in the Union will afford increased bathing facilities to the masses of the people and thus vastly improve the public health.

